# Pregnancy outcomes in infertile patients with endometrial hyperplasia with or without atypia undergoing *in vitro* fertilization: the early-follicular long protocol is superior to midluteal long protocol

**DOI:** 10.3389/fendo.2024.1314432

**Published:** 2024-02-19

**Authors:** Huiling An, Tongjie Li, Kai Huang, Hao Shi, Chen Wang, Ting Chu, Jun Zhai

**Affiliations:** ^1^ Center for Reproductive Medicine, The First Affiliated Hospital of Zhengzhou University, Zhengzhou, China; ^2^ Henan Key Laboratory of Reproduction and Genetics, The First Affiliated Hospital of Zhengzhou University, Zhengzhou, China; ^3^ Henan Provincial Obstetrical and Gynecological Diseases (Reproductive Medicine) Clinical Research Center, The First Affiliated Hospital of Zhengzhou University, Zhengzhou, China

**Keywords:** endometrial hyperplasia, early-follicular, midluteal, long protocol, *in vitro* fertilization, pregnancy outcomes

## Abstract

**Background:**

Although *in vitro* fertilization (IVF) in infertile patients with endometrial hyperplasia is common after drug treatment, the pregnancy outcomes are often unsatisfactory. Till date, no studies have reported the outcome of patients with endometrial hyperplasia treated using early-follicular long (EL) protocol and midluteal long (ML) protocol.

**Objective:**

To evaluate the pregnancy outcomes and disease prognosis of patients with endometrial hyperplasia with or without atypia undergoing IVF treatment with EL protocol or ML protocol.

**Methods:**

This was a retrospective study in university-affiliated reproductive medical center. A total of 138 patients with endometrial hyperplasia undergoing IVF treatment were included to compare the pregnancy outcomes and disease prognosis between EL and ML protocols. We further matched 276 patients with normal endometrium to compare the pregnancy outcomes between patients with endometrial hyperplasia and patients with normal endometrium under different controlled ovarian stimulation (COS) protocol.

**Results:**

In patients with endometrial hyperplasia, the clinical pregnancy rate (CPR) and live birth rate (LBR) were significantly higher in EL protocol than in ML protocol (61.8% vs. 43.5%, P=0.032; 50.0% vs. 30.6%, P= 0.022). In the ML protocol, patients with endometrial hyperplasia had significantly lower CPR and LBR than those with normal endometrium (43.5% vs. 59.7%, P=0.037; 30.6% vs. 49.2%, P=0.016). While in the EL protocol, they achieved similar CPR and LBR as patients with normal endometrium (61.8% vs. 69.7%, P=0.232; 50.0% vs. 59.9%, P=0.156). In patients with endometrial hyperplasia, COS protocol was an independent factor affecting clinical pregnancy (adjusted odds ratio [OR] 2.479; 95% confidence interval [CI] 1.154-5.327) and live birth (adjusted OR 2.730; 95% CI 1.249-5.966). After 1–10 years of follow-up, no significant difference was found in the recurrence rate of endometrial lesions between both treatment groups.

**Conclusions:**

For patients with endometrial hyperplasia undergoing IVF treatment, the EL protocol is superior to ML protocol, and in the EL protocol, they can achieve similar pregnancy outcomes as patients with normal endometrium.

## Introduction

Endometrial hyperplasia is defined as irregularity and cystic expansion of glands (simple) or crowding and budding of glands (complex) without worrisome changes in the appearance of individual gland cells (1, 2). The underlying cause of the hyperplasia is a relative predominance of estrogen combined with insufficient progesterone levels (2). In 2014, based on the presence or absence of cellular atypia, endometrial hyperplasia was categorized into two types by the World Health Organization: endometrial hyperplasia without atypia (EH) and endometrial atypical hyperplasia (EAH) (1). EAH is a recognized precancerous condition called endometrial intraepithelial neoplasia (EIN) (1, 3). The frequency of endometrial hyperplasia found in infertile women undergoing *in vitro* fertilization (IVF) treatment for the first time is approximately 3% (4). Prior studies have reported that complete remission of endometrial hyperplasia can be achieved in up to 80% - 90% patients using high-efficiency progesterone treatment (5, 6).

Patients with endometrial hyperplasia complicated with infertility often need assisted reproductive technology (ART) for achieving pregnancy, but studies found that irrespective of the use of midluteal long (ML) protocol or gonadotropin-releasing hormone (GnRH) antagonist protocol for controlled ovarian stimulation (COS), the pregnancy outcomes of the patients with endometrial hyperplasia were significantly lower than those of the patients with normal endometrium (7, 8). At the same time, due to concerns that the increase in estrogen (E2) level during COS would increase the risk of recurrence of endometrial lesions, Azim et al. (9) suggested to choose the mild stimulation protocol or the combination of letrozole during COS to reduce E2 level. However, the mild stimulation protocol usually resulted in fewer oocytes retrieved and embryos available. For protecting the endometrium, Chen et al. adopted progestin primed ovarian stimulation (PPOS) protocol, and compared the curative effect with the mild stimulation protocol in the freeze-thaw cycle (10). They found that PPOS protocol provided higher clinical pregnancy rate (CPR) compared to mild stimulation protocol (40.54%vs. 9.38%) (10). However, the disadvantage of the PPOS protocol is that fresh embryo transfer cannot be performed, which increases the economic burden of the patients and delays the time for the patients to reach pregnancy. Therefore, further research is urgently needed to explore the optimal COS protocol for these patients.

In recent years, because the early-follicular long (EL) protocol has shown better pregnancy outcomes and can reduce the risk of ovarian hyperstimulation syndrome (OHSS), it has gradually become the predominant choice in many reproductive centers in China. Xu et al. (11) showed that in the normal population, the LBR of EL protocol was significantly higher than that of ML protocol and GnRH antagonist protocol in fresh embryo transfer cycles (62.6% vs. 52.1% vs. 45.6%). In the process of COS, since the E2 level on trigger day was lower in the EL protocol than ML protocol, it may be more beneficial to patients with endometrial hyperplasia. To the best of our knowledge, till date, no studies have reported pregnancy outcomes of patients with endometrial hyperplasia treated using EL protocol. This study retrospectively collected the data of infertile patients with endometrial hyperplasia undergoing IVF/ICSI treatment in the Reproductive Medicine Center of our hospital, and compared the effects of EL protocol and ML protocol on pregnancy outcomes and disease prognosis, to optimize a COS protocol for these patients.

## Materials and methods

### Study design

In this retrospective cohort study, we analyzed the clinical data of patients undergoing their first IVF cycle at Reproductive Medicine Center of the public university hospital between January 2011 and January 2021. This research was approved by Institutional Review Board (2023-KY-0236-001). The requirement for informed consent was waived due to the retrospective character of this study.

Inclusion criteria: 1. age 20–40 years, 2. underwent the first IVF cycle and fresh embryo transfer. Exclusion criteria: 1. uterine malformation; 2. uterine submucosal fibroids; 3. intrauterine adhesions; 4. endometriosis or adenomyosis; 5. serious endocrine diseases; 6. untreated hydrosalpinx; 7. preimplantation genetic testing, and 8. severe oligozoospermia or teratozoospermia in the male partner.

**Figure 1:**
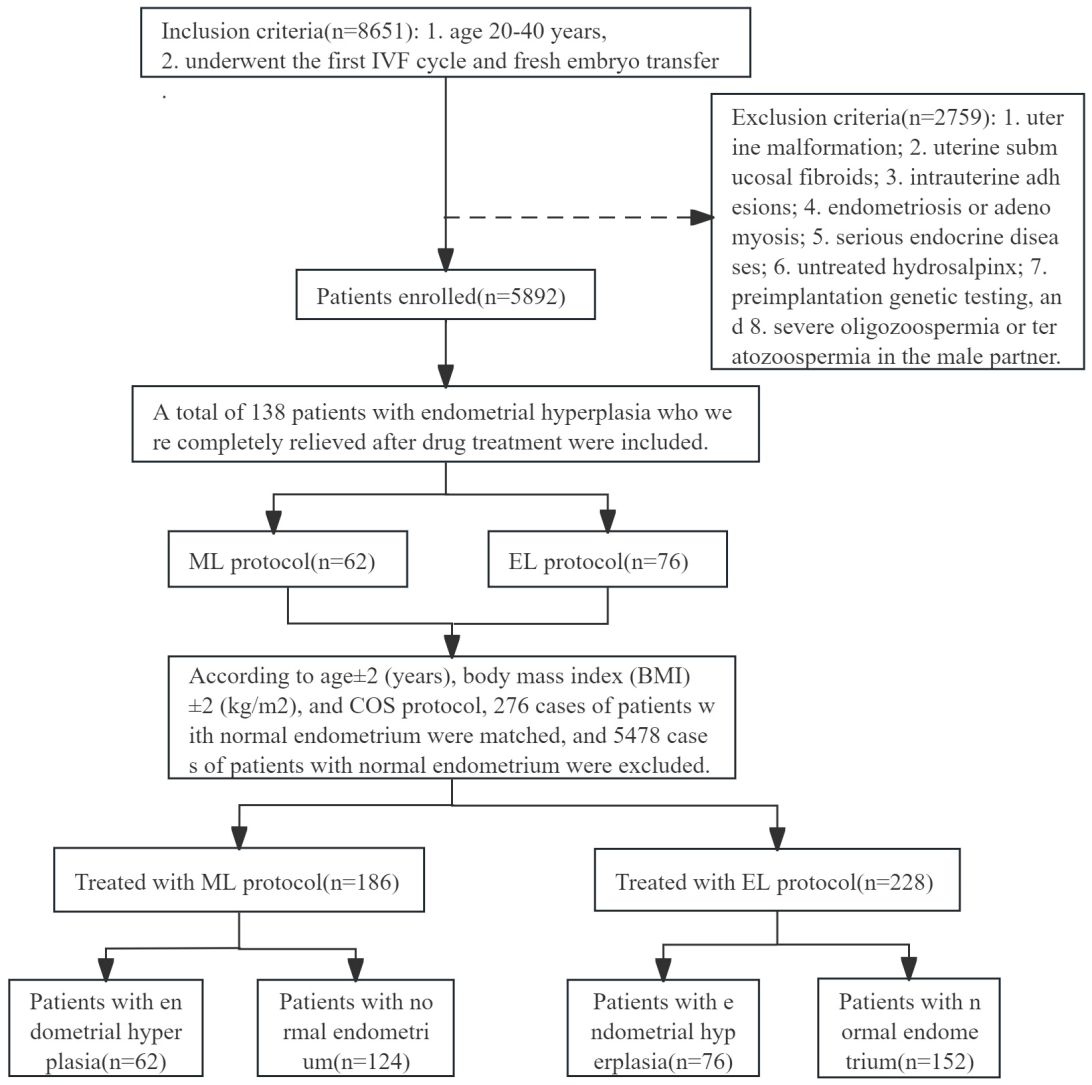
Flowchart describing the study population.

A total of 138 patients with endometrial hyperplasia (EH: 114 cases; EAH/EIN: 24 cases) who were completely relieved after drug treatment were included to compare the pregnancy outcomes and disease prognosis between EL and ML protocols. According to age ± 2 (years), body mass index (BMI) ± 2 (kg/m^2^), and COS protocol, we further matched 276 patients with normal endometrium to compare the pregnancy outcomes between patients with endometrial hyperplasia and patients with normal endometrium under different COS protocol. We defined patients with normal endometrium based on normal morphological diagnosis under hysteroscopy and normal pathological reports after endometrial biopsy. The flow chart of the study is presented in **Figure 1**.

### Endometrial hyperplasia reversal treatment

Patients with EH were prescribed megestrol acetate (Qingdao Guohai Biological Pharmaceutical Co., Ltd., China), 160 mg/day orally, and ultrasound examination and endometrial biopsy were performed every 6 months during treatment until no endometrial lesions was found in two consecutive endometrial biopsies (12). Patients with EAH/EIN were treated with lesion resection under the guidance of hysteroscopy, followed by treatment with levonorgestrel intrauterine system (Mirena; Bayer Oy) and/or megestrol acetate (160–320 mg/day), and ultrasound examination and endometrial biopsy were performed every 3 months during treatment until no endometrial lesions was found in two consecutive endometrial biopsies (13). Patients were transferred to a reproductive center for treatment after complete remission.

### IVF protocols

#### Downregulation regimen

EL protocol: On the 2^nd^ or 3^rd^ day of menstruation, patients were administered once 3.75 mg of the long-acting GnRH agonist (Diphereline; Beaufour Ipsen, Dreux, France). Serum follicle-stimulating hormone (FSH), luteinizing hormone (LH), estrogen (E2), and progesterone (P) were measured 28 days after injection. Follicle size was monitored using transvaginal ultrasound. ML protocol: On the 21^st^ or 22^nd^ day of the menstrual cycle, patients were administered 0.1 mg of the short-acting GnRH agonist (Triptorelin; IPSEN Biotechnology, France) for 14–16 days. Serum FSH, LH, E2, and P were measured after injection, meanwhile follicular size was monitored by vaginal ultrasound.

#### Ovulation stimulation

Gonadotropin (Gn) (Gonal-f; Merck Serono, Darmstadt, Germany) was used to promote multiple synchronized development and maturation of follicles once the downregulation criteria were reached in both protocols with serum FSH level suppressed to <5 mIU/mL, LH level <3 mIU/mL, E2 level <50 pg/mL, antral follicle diameter about 3–5 mm, and no ovarian cysts with diameter >10 mm. The dosage of Gn was adjusted in accordance with the blood hormone levels and follicle size.

#### Human chorionic Gn injection and oocyte retrieval

When the diameter of three dominant follicles were ≥18 mm or the proportion of follicles with diameters >16 mm accounted for more than two-thirds of the total follicles, hCG (2000 IU, Livzon Pharmaceutical, China) were administered. Oocytes were extracted by puncture under the guidance of vaginal ultrasound at 36-37 h after the hCG trigger injection.

#### Embryo transfer

Embryo transfer was carried out 3-5 days following oocyte retrieval according to the embryo quality, endometrial status, and general health of the patients.

### Follow-up

Follow-up was conducted through telephone until June 2022. Pregnancy outcomes, neonatal weight, and recurrence of endometrial lesions over 1–10 years were collected.

### Clinical outcomes

Pregnancy-induced hypertension (PIH): new onset of hypertension after 20 weeks of pregnancy (14); Low birth weight (LBW): birth weight less than 2,500 g; Recurrence of endometrial lesions: the reappearance of EH, EAH/EIN, or progression to endometrial carcinoma during IVF treatment or follow-up after IVF. Implantation rate (number of gestational sacs observed/number of embryos transplanted×100%); CPR (number of clinical pregnancies/number of transplant cycles×100%); LBR (number of deliveries that resulted in at least one live born baby/number of transplant cycles×100%) (15). Adverse pregnancy outcomes include abortion, preterm birth (PTB), gestational diabetes (GDM), PIH, premature rupture of membranes (PROM) (16); Adverse pregnancy outcomes rate (number of women with adverse pregnancy outcomes/number of clinical pregnancies×100%); LBW rate (number of deliveries with at least one LBW infant/number of deliveries×100%) (17, 18).

### Statistical analysis

SPSS 25 (IBM Corp. Released 2017. IBM SPSS Statistics for Windows, Version 25.0. Armonk, NY: IBM Corp) was used for all analyses. Categorical variables are given as percentages, whereas continuous variables are expressed as the mean ± standard deviation (x ± s). To compare differences in the mean values of continuous variables, the t-test was used, while the Chi-square (X^2^) test was used to compare categorical variables. Univariate logistic regression was performed on variables that may affect clinical pregnancy and live birth in patients with endometrial hyperplasia, and multivariate logistic regression was performed on variables found to be significant after univariate logistic regression and variables considered significant in previous literature. The threshold for statistical significance was set at P<0.050.

## Results

### Comparison of patients’ general characteristics

In patients with endometrial hyperplasia, the proportion of blastocyst stage embryo was significantly higher in EL protocol than in ML protocol (25.0%vs. 11.3%, P=0.040). In the EL protocol, the basal E2 level and the proportion of primary infertility were significantly higher in patients with endometrial hyperplasia than in patients with normal endometrium (51.85 ± 31.29vs. 39.69 ± 29.04 pg/ml, P=0.005; 68.4%vs. 54.6%, P=0.045) (**Table 1**).

**Table 1:** Comparison of patients’ general characteristics.

	Endometrial hyperplasia			Normal Endometrium		Normal Endometrium		
	ML protocol(n=62)	EL protocol(n=76)	P value^a^	ML protocol(n=124)	P value^b^	EL protocol (n=152)	P value^c^	
Age(year)	30.85±4.10	31.32±3.90	0.501	30.96±4.11	0.870	31.48±3.82	0.761	
BMI (kg/m^2^)	24.02±3.02	24.88±3.04	0.102	24.12±2.84	0.827	24.55±2.87	0.425	
Basal FSH (mIU/ml)	6.51±1.76	6.18±2.15	0.336	6.68±1.73	0.529	6.45±1.51	0.333	
Basal LH (mIU/ml)	4.58±1.73	4.39±2.08	0.559	4.73±1.93	0.607	4.41±1.32	0.949	
Basal E2 (pg/ml)	49.62±25.20	51.85±31.29	0.651	42.13±24.17	0.051	39.69±29.04	0.005	
AFC(n)	11.63±7.46	12.68±6.96	0.392	11.35±6.87	0.798	12.78±6.11	0.913	
Duration of infertility (year)	4.73±2.95	4.37±2.62	0.453	4.88±2.20	0.691	4.07±2.63	0.414	
Type of infertility			0.457		0.183		0.045
Secondary infertility (%)	25.8% (16/62)	31.6% (24/76)		35.5% (44/124)		45.4% (69/152)		
Primary infertility (%)	74.2% (46/62)	68.4% (52/76)		64.5% (80/124)		54.6% (83/152)		
Histological type			0.209		/		/	
EH (%)	87.1% (54/62)	78.9% (60/76)		/		/		
EAH/EIN (%)	12.9% (8/62)	21.1% (16/76)		/		/		
Type of embryo transferred			0.040		0.377		0.247	
Cleavage stage embryo (%)	88.7% (55/62)	75.0% (57/76)		83.9% (104/124)		81.6% (124/152)		
Blastocyst stage embryo (%)	11.3% (7/62)	25.0% (19/76)		16.1% (20/124)		18.4% (28/152)		
No. of embryos transferred (n)	1.82±0.56	1.66±0.48	0.064	1.87±0.57	0.583	1.78±0.41	0.054	

Continuous data: mean ± SD. Categorical data: % (n/N). BMI, body mass index; FSH, follicle-stimulating hormone; LH, luteinizing hormone; E2, estradiol; AFC, antral follicle count; EH, endometrial hyperplasia without atypia; EAH/EIN, endometrial atypical hyperplasia/endometrial intraepithelial neoplasia. ^a^P value for Endometrial hyperplasia-ML protocol vs. Endometrial hyperplasia-EL protocol. ^b^P value for Endometrial hyperplasia-ML protocol vs. Normal endometrium -ML protocol. ^c^P value for Endometrial hyperplasia-EL protocol vs. Normal endometrium -EL protocol. The significance was considered at p<0.05.

### Comparison of laboratory parameters and clinical outcomes

In patients with endometrial hyperplasia, the implantation rate, the CPR and LBR were significantly higher in EL protocol than in ML protocol (49.2%vs. 31.9%, P=0.006; 61.8% vs. 43.5%, P=0.032; 50.0% vs. 30.6%, P= 0.022). There was no significant difference in the number of oocytes retrieved between EL and ML protocols, but the E2 level and LH level on trigger day of EL protocol were significantly lower (2133.91 ± 1329.37vs. 4152.90 ± 2431.81 pg/ml, P=0.001; 0.73 ± 0.68mIU/mL vs. 1.83 ± 0.87mIU/mL, P=0.001). In the ML protocol, patients with endometrial hyperplasia had significantly lower CPR and LBR than those with normal endometrium (43.5% vs. 59.7%, P=0.037; 30.6% vs. 49.2%, P=0.016). While in the EL protocol, patients with endometrial hyperplasia achieved similar CPR and LBR as patients with normal endometrium (61.8% vs. 69.7%, P=0.232; 50.0% vs. 59.9%, P=0.156). No matter in ML or EL protocol, the endometrial thickness on trigger day of patients with endometrial hyperplasia was significantly thinner than that of patients with normal endometrium (11.26 ± 2.08vs. 12.50 ± 2.74 mm, P=0.001; 11.43 ± 2.48vs. 12.73 ± 2.48 mm, P=0.001). No significant differences were found in terms of the number of high-quality embryos, adverse pregnancy outcomes rate, neonatal weight, or recurrence rate of endometrial lesions (**Table 2**).

**Table 2:** Comparison of laboratory parameters and clinical outcomes.

	Endometrial hyperplasia			Normal endometrium		Normal endometrium	
	ML protocol (n=62)	EL protocol(n=76)	P value^a^	ML protocol (n=124)	P value^b^	EL protocol (n=152)	P value^c^
Total dosage of Gn used (IU)	2217.14±831.20	3045.56±1240.17	0.001	2304.84±760.57	0.473	2927.63±934.74	0.466
Length of Gn used(day)	11.79±2.17	13.63±2.62	0.001	12.06±1.75	0.354	13.87±2.25	0.480
No. of oocytes retrieved (n)	10.58±5.71	11.03±5.99	0.658	10.28±4.81	0.709	12.09±5.87	0.201
No. of 2PN fertilization (n)	7.13±4.35	6.84±3.93	0.685	6.84±3.60	0.630	7.59±4.19	0.199
No. of high-quality embryos (n)	5.03±3.28	4.41±3.29	0.269	4.71±2.70	0.505	4.97±2.88	0.189
Endometrial thickness on trigger day (mm)	11.26±2.08	11.43±2.48	0.657	12.50±2.74	0.001	12.73±2.48	0.001
E2 level on trigger day (pg/ml)	4152.90±2431.81	2133.91±1329.37	0.001	4413.98±3070.86	0.560	2731.93±1389.15	0.002
LH level on trigger day (mIU/ml)	1.83±0.87	0.73±0.68	0.001	1.96±0.95	0.358	0.75±0.65	0.777
Implantation rate (%)	31.9% (36/113)	49.2% (62/126)	0.006	39.7% (92/232)	0.159	50.6% (137/271)	0.803
Biochemical pregnancy rate (%)	53.2% (33/62)	64.5% (49/76)	0.181	63.7% (79/124)	0.168	71.7% (109/152)	0.264
Clinical pregnancy rate (%)	43.5% (27/62)	61.8% (47/76)	0.032	59.7% (74/124)	0.037	69.7% (106/152)	0.232
Live birth rate (%)	30.6% (19/62)	50.0% (38/76)	0.022	49.2% (61/124)	0.016	59.9% (91/152)	0.156
Ectopic pregnancy rate (%)	3.7% (1/27)	0	–	1.4% (1/74)	1.000	0	–
adverse pregnancy outcomes rate (%)	44.4% (12/27)	34.0% (16/47)	0.374	35.1% (26/74)	0.393	31.1% (33/106)	0.722
Abortion rate (%)	25.9% (7/27)	19.1% (9/47)	0.495	16.2% (12/74)	0.269	14.2% (15/106)	0.433
PTB rate (%)	11.1% (3/27)	14.9% (7/47)	0.916	10.8% (8/74)	1.000	15.1% (16/106)	0.974
GDM rate (%)	0	2.1% (1/47)	–	5.4% (4/74)	0.512	3.8% (4/106)	0.972
PIH rate (%)	7.4% (2/27)	4.3% (2/47)	0.965	4.1% (3/74)	0.866	0.9% (1/106)	0.465
PROM rate (%)	7.4% (2/27)	2.1% (1/47)	0.620	2.7% (2/74)	0.620	2.8% (3/106)	1.000
Neonatal weight(g)	3273.68±418.37	3182.76±528.85	0.516	3364.81±579.62	0.528	3235.01±649.88	0.662
LBW rate (%)	15.8% (3/19)	15.8% (6/38)	1.000	11.5% (7/61)	0.921	12.1% (11/91)	0.571
recurrence rate of endometrial lesions (%)	16.1% (10/62)	9.2% (7/76)	0.219	/	/	/	/

Continuous data: mean ± SD. Categorical data: % (n/N). Gn, gonadotropin; d, days; 2PN, 2 pronuclei; E2, estradiol; LH, luteinizing hormone; adverse pregnancy (including abortion, PTB, GDM, PIH, and PROM); PTB, preterm birth; GDM, gestational diabetes; PIH, pregnancy-induced hypertension; PROM, Premature rupture of membranes; LBW, low birth weight. ^a^P value for Endometrial hyperplasia-ML protocol vs. Endometrial hyperplasia-EL protocol. ^b^P value for Endometrial hyperplasia-ML protocol vs. Normal endometrium -ML protocol. ^c^P value for Endometrial hyperplasia-EL protocol vs. Normal endometrium -EL protocol. The significance was considered at p<0.05.

### Logistic regression

**Figure 2:**
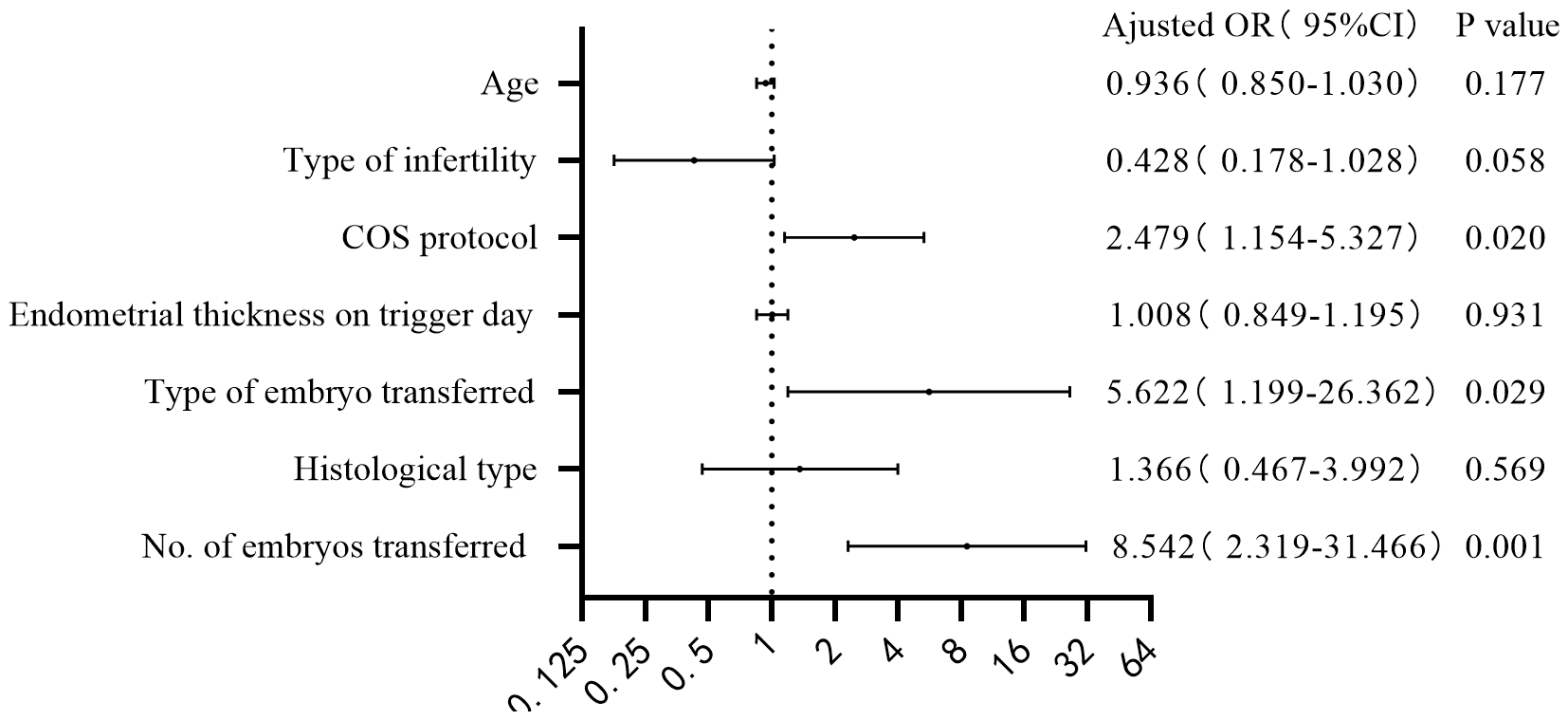
Forest plot: multivariate logistic regression performed for clinical pregnancy.

**Figure 3:**
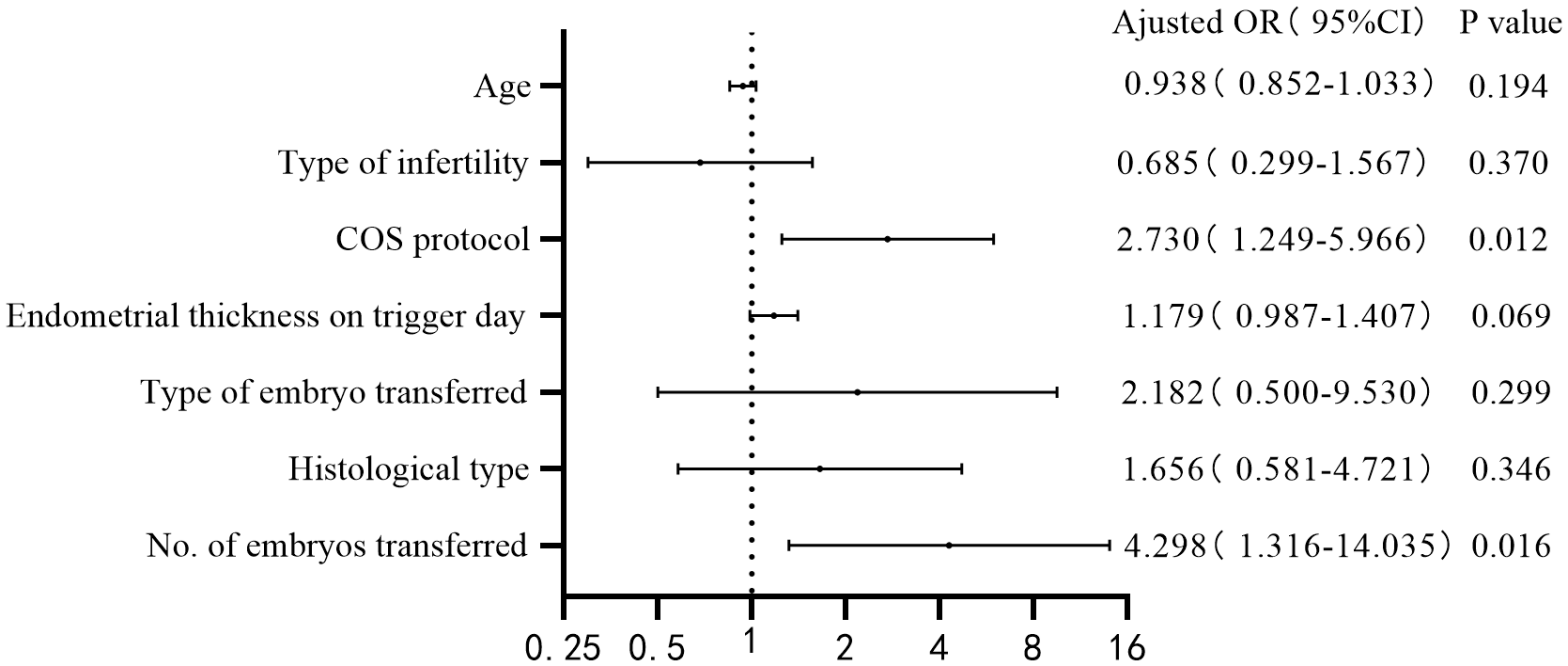
Forest plot: multivariate logistic regression performed for live birth.

Age, type of infertility (reference: secondary infertility), COS protocol (reference: ML protocol), endometrial thickness on trigger day, type of embryo transferred (reference: cleavage stage embryo), histological type (reference: EH), and number of embryos transferred were included for the multivariate logistic regression. The multivariate logistic regression results are shown in **Figures 2**, **3**. COS protocol (adjusted odds ratio [OR] 2.479; 95% confidence interval [CI] 1.154-5.327), type of embryo transferred (adjusted OR 5.622; 95% CI 1.199-26.362), and number of embryos transferred (adjusted OR 8.542; 95% CI 2.319-31.466) were independent factors affecting clinical pregnancy. COS protocol (adjusted OR 2.730; 95% CI 1.249-5.966), number of embryos transferred (adjusted OR 4.298; 95% CI 1.316-14.035) were independent factors affecting live birth.

### Recurrence in patients with endometrial hyperplasia after complete remission

Seven of 76 patients in the EL protocol relapsed, with a recurrence rate of 9.2% (7/76). Ten of 62 patients in the ML protocol relapsed, with a recurrence rate of 16.1% (10/62). Eleven of 114 EH patients relapsed, with a recurrence rate of 9.6% (11/114). Six of 24 EAH/EIN patients relapsed, with a recurrence rate of 25.0% (6/24). After the relapse of eleven patients with EH, eight patients remained as endometrial hyperplasia without atypia, while three patients progressed to endometrial atypical hyperplasia/endometrial intraepithelial neoplasia. After the relapse of six patients with EAH/EIN, three patients remained as endometrial atypical hyperplasia/endometrial intraepithelial neoplasia, two patients were endometrial hyperplasia without atypia, and one patient progressed to carcinoma.

## Discussion

The risk factors of endometrial lesions are also common causes of infertility. Since patients with endometrial hyperplasia are often accompanied by ovulation disorders, the proportion of primary infertility among such patients in this study was as high as 71.0%. At the same time, endometrial lesions, in turn, would have a negative effect on embryo implantation, thereby aggravating infertility.

Although ART assisted pregnancy in patients with endometrial hyperplasia is common after drug treatment, the pregnancy outcomes are often unsatisfactory (7, 8, 19, 20). Fujimoto et al. (7) showed that when using ML protocol for COS, the CPR was significantly lower in patients with EAH/EIN than patients with normal endometrium (9.5%vs. 35.7%). This study showed that, in the ML protocol, the CPR and LBR of patients with endometrial hyperplasia were significantly lower than those of patients with normal endometrium (43.5% vs. 59.7%; 30.6% vs. 49.2%), consistent with the findings of Fujimoto et al. In another study, which investigated the GnRH antagonist protocol for COS, patients with endometrial hyperplasia also had significantly lower LBR than those with normal endometrium (8). The possible reasons for this result are as follows:1. High-efficiency progesterone can cause a reduction in the number of glandular cells in the endometrium and the endometrium atrophy (21, 22). 2. During drug therapy, frequent endometrial scrapings are required to assess the effect of treatment, which may lead to endometritis and thinning of endometrial damage in some patients (22, 23).

Selecting the best COS protocol for patients with endometrial hyperplasia and improving the pregnancy outcomes of these patients warrants clinical attention. Due to its higher CPR and lower risk of OHSS compared to other protocols, the EL protocol is more widely used in current clinical practice (11, 24, 25). Therefore, for the first time, we investigated the pregnancy outcomes of infertile patients with endometrial hyperplasia using EL protocol for COS, and compared them with the outcomes of the ML protocol. The results showed that, in patients with endometrial hyperplasia, treatment using EL protocol provided higher CPR and LBR compared to the ML protocol (61.8% vs. 43.5%; 50.0% vs. 30.6%). This study found that the number of oocytes retrieved and high-quality embryos were similar between two COS protocols, so the improvement in pregnancy outcomes of patients using EL protocol is more likely due to the increased endometrial receptivity. Due to the administration of long-acting GnRH agonist, the EL protocol provides the endometrium with a longer rest time, which may be beneficial to the recovery of endometrial tissue function (26). Studies have revealed that the expression of endometrial receptivity markers was significantly higher in patients treated with EL protocol than patients treated with ML protocol, including homeobox A10 and leukemia inhibitory factor (11). In the EL protocol, patients with endometrial hyperplasia achieved similar CPR and LBR as patients with normal endometrium (61.8% vs. 69.7%; 50.0% vs. 59.9%), which may be attributed to the improvement of endometrium by the long-acting GnRH agonist.

Current study shows that ART treatment does not increase the risk of recurrence of endometrial lesions (27, 28). In this study, the recurrence rate of patients with EAH/EIN after ART treatment was 25%, which was similar to the recurrence rate of endometrial lesions (26%-32.4%) without ART for assisted pregnancy (29–31). Additionally, patients in the EL protocol were administered the long-acting GnRH agonist, resulting in increased suppression of LH. The LH level on trigger day is significantly lower in the EL protocol than that in the ML protocol (0.73 ± 0.68 mIU/mL vs. 1.83 ± 0.87 mIU/mL, P=0.001). According to the classic bicellular bigonadal hormone theory, the relative deficiency of LH weakens the process of estrogen synthesis in granulosa cells, resulting in a lower level of E2 on trigger day in the EL protocol and less stimulation to the endometrium, which enhances safety of patients with endometrial hyperplasia.

This study comprehensively compared the pregnancy outcomes, perinatal outcomes, and disease prognosis of patients with endometrial hyperplasia treated with EL protocol and ML protocol for the first time. The results suggest that the EL protocol maybe a better COS protocol for patients with endometrial hyperplasia, which has certain guiding significance for the selection of COS protocols for patients with endometrial hyperplasia. The limitation of this study is that this is a retrospective study conducted in a single center. The results of our study need to be confirmed by large-sample, multi-center, and prospective studies in the future.

## Conclusions

For patients with endometrial hyperplasia undergoing IVF treatment, the EL protocol is superior to ML protocol, and in the EL protocol, they can achieve similar pregnancy outcomes as patients with normal endometrium.

## Data availability statement

The datasets presented in this study can be found in online repositories. The names of the repository/repositories and accession number(s) can be found below: DOI: 10.5281/zenodo.10417109.

## Ethics statement

This retrospective cohort study was approved by the Ethics Review Committee of the First Affiliated Hospital of Zhengzhou University (2023-KY-0236-001). The studies were conducted in accordance with the local legislation and institutional requirements. Written informed consent for participation was not required from the participants or the participants’ legal guardians/next of kin because The requirement for informed consent was waived due to the retrospective character of this study.

## Author contributions

HA: Data curation, Investigation, Methodology, Writing –original draft. TL: Investigation, Writing – review & editing. KH: Writing – review & editing. HS: Writing – review & editing. CW: Writing – review & editing. TC: Writing – review & editing. JZ: Conceptualization, Writing – review & editing.
